# Finding my ground in public health research: lessons from my Grandmother’s kitchen

**DOI:** 10.1186/1471-2458-11-S5-S2

**Published:** 2011-11-25

**Authors:** Tanya Koolmatrie

**Affiliations:** 1 Healthy Mothers Healthy Families Research Group, Murdoch Childrens Research Institute, Level 1, 369 Royal Pde, Parkville VIC 3052, Australia

## Abstract

**Background:**

Research has a 'bad name' in Aboriginal communities. Too often, researchers have come, gathered information and taken it away from Aboriginal people, with no benefit for the communities taking part in the research. This history has implications for Aboriginal and non-Aboriginal researchers planning research with Aboriginal communities. An in depth interview study will be conducted in one region of Victoria. Participants will be Aboriginal women who have had a baby within the previous five years. Processes that have been used in preparing to 'step out' into the community to conduct this research are the focus of the paper.

## Key reflections

Before stepping out into an Aboriginal community as an Aboriginal researcher to interview women and listen to their stories, other processes need to be put in place. These processes include establishing support within the community for the research to be undertaken and to establish ways for Aboriginal people within the community to be involved in the research. This begins with the building of relationships. Learning how to do this began in my Grandmother's kitchen, where making cups of tea for Elders and learning when and how to listen, was the vehicle for teaching me about how to build relationships; about my cultural roles and responsibilities; and about cultural protocols and when and how to use them. Lessons learned in my Grandmother's kitchen are also helping me to navigate the complexity of being both an 'insider' and an 'outsider' in the Aboriginal community where the research is being undertaken.

### Introduction

There is mounting evidence to show that Aboriginal and Torres Strait Islander women and other Indigenous women experience greater psychological distress; higher rates of trauma, grief and loss; and worse mental health than non-Indigenous women [1-6]. Recent Australian studies provide evidence that Aboriginal and Torres Strait Islander women are more likely than non Indigenous women to experience depression in the postnatal period [2,4]. However, the research literature on this area is limited, and in the main, has not been undertaken in ways that are consistent with recent guidelines for research involving Aboriginal people [7].

### Getting started as an Aboriginal researcher

My research focuses on the social and emotional wellbeing of Aboriginal women during pregnancy and the early postnatal period. My own experiences of becoming and being a mother and knowledge of what it means to be a mother in Aboriginal communities have shaped and informed the way I am conceptualising and planning my research. My main method of data collection will be in-depth interviews with Aboriginal women who have been pregnant or had a pregnancy in the last five years. The focus of the interviews will be on women’s experiences and understandings of factors influencing maternal social and emotional wellbeing in the antenatal and postnatal periods.

The setting for my research is a regional city in Victoria that includes a diverse Aboriginal community. I will be recruiting and collecting data in a community where I am potentially known to participants. I also have community and familial ties within this setting. This presents both challenges and rewards. Maintaining trust, rigour, and integrity and most importantly undertaking my research in ways that are respectful, reciprocal and in consultation with the Aboriginal community are essential elements in planning and carrying out Aboriginal health research [7-10].

Building trustful relationships, consulting with community members, boards and staff members of community organisations are part of the research process.

Research in any setting is challenging. As an Aboriginal researcher living in the community where I plan to carry out my research, there are some particular challenges. The relationships I am building now with the community and the impact of the research I undertake will continue during and after my study is complete. They will have flow on effects into my life well after the research is over [11]. There may be an impact on relationships with individuals who choose or choose not to participate in the research. The way that I am viewed by my community may change. The way that I view myself in my community may change. These are risks and challenges that Aboriginal researchers take in order to undertake research in their own communities. A dilemma for Aboriginal researchers is how great the risk of changing relationships is, and will these changes be for better, will they result in benefits to communities that outweigh any costs incurred in doing the research.

### Moving on from past mistakes

Research has a ‘bad name’ in Aboriginal communities [12]. Historically, research was undertaken in ways that denied and excluded Aboriginal people from having a voice in the research process. Indigenous Maori academic, Linda Tuhawai Smith writes that the word ‘research’ is probably one of the dirtiest words in the Indigenous world’s vocabulary. When mentioned in many Indigenous contexts, it stirs up silence, it conjures up bad memories, and it raises a smile of knowing that is distrustful. Such reactions stem from experiences of being treated as ‘objects’ for investigation. On too many occasions researchers viewed Aboriginal knowledge as something to gather and take away from Aboriginal people. Research (in its many forms) has denied Aboriginal people the right to sovereignty in relation to the design and conduct of research, interpretation of findings and dissemination of results [7-10,13].

The call from many quarters for changes to past practices of exclusion of Aboriginal people in the shaping, conduct and interpretation of research has meant looking at research processes from a different perspective, learning from the mistakes of the past, and mapping out new approaches. Guidelines for Indigenous health research were published by the Australian National Health and Medical Research Council in 2003 [7]. This document reinforces the importance of core values and ethics for research in Indigenous communities. A companion report called *Keeping Research on Track: A guide for Aboriginal and Torres Strait Islander Peoples about Research Ethics* states that “Aboriginal and Torres Strait Islander peoples have a right, and indeed a responsibility, to be involved in all aspects of research undertaken in our communities” and highlights the importance of research respecting “the shared values, diversity, priorities, needs and aspirations of Aboriginal and Torres Strait Islander peoples, as well as researchers and other Australians” [8]. This approach focuses on the ways that research can be undertaken in respectful, reciprocal ways that involve the Aboriginal community at all stages of the research process, beginning with the building of relationships. These principles, values and ethics that are the cornerstone of Aboriginal health research will govern the way my research will be undertaken.

### Learning how and when to listen

I am Ngarrindjeri. I was raised on Raukkan, which lies on the shores of Lake Alexandrina in South Australia. I consider myself privileged to have had the childhood that I did. I consider myself incredibly lucky. I had the privilege of being raised by my Grandmother and other Elders, Aunties and extended family.

My Grandmother’s kitchen was the place where I began to learn many of the skills I am now using in my research. Much of my childhood was spent in my Grandmother’s kitchen surrounded by Elders, Aunties and other extended family.

It is here, in my Grandmother’s kitchen where the learnings I now bring to my research began. I used to make cups of tea for my Grandmother, Aunties and Elders. This was a vehicle used to teach me lessons about listening, how to listen, when to listen and when not to. Essentially, I was being taught how to build relationships through cultural knowledge and understanding. The most prestigious times in my childhood were when an empty seat was pulled away from my Grandmother’s kitchen table. This meant that I had a place, a place at the table with my Elders. These were times of learning and listening to stories being shared about our culture and history. I learnt of the importance of my cultural identity through story telling. I also learnt of the power of story, and about when and how to pass on knowledge in this way.

Story-telling is a central aspect of Aboriginal culture and has been for thousands of years. Tuhawai Smith has written about the importance of the perspectives of Elders in Indigenous cultures, and about the way in which individual stories contribute to a collective story, which is fundamental to the way(s) that Aboriginal women talk to each other, largely by story-telling [13]. The interplay of many skills is being exchanged during this process and includes more than simply sharing information. Mindell also writes about the use of story-telling by Indigenous peoples around the world as a valuable means of healing [14]. Mindell recognises the strength of story-telling as a means of communication and emphasises its potential as a launching pad for reconciliation when communication breaks down. Historically, Australian Aboriginal people have survived colonialist attempts to steal, forbid and demonise our stories. Despite such attempts, we have maintained our story-telling skills along with our stories.

I have been privileged in my life to have been exposed to this part of our culture from a very early age. This applies to me as an Aboriginal researcher on many levels. By listening to what stories are being told to me by Aboriginal women in the community, I am reminded of my cultural background and of protocols taught to me in my Grandmother’s kitchen. Stories told to me also enrich the ways in which I am going about my research. This is an incredible privilege. To hear the voices of Aboriginal women and apply their wisdom to the ways that I go about my research means that I am in a position to shape my research in ways that matter to Aboriginal women and to implement methods that will make a contribution to knowledge about Aboriginal women’s wellbeing during and after pregnancy. One approach I try to apply in the way that I go about my research is to listen to what story is being told to me, go away and think about why that story was told to me at that particular time and what messages and teachings were in the story, that I am meant to learn from, to hear and to go forward with.

### Learning how to apply cultural knowledge to research

Those days in my Grandmother’s kitchen taught me things that I have brought into the world of public health research. I am now navigating my way through the exchange and interplay of the knowledge systems of my heritage and academic ways of thinking. Trying to marry the two different types of knowledge systems is challenging. Weaving my cultural protocols and complexities of public health research into the framework of an academic PhD is exciting and presents opportunities for approaches to public health research that bring to life principles, guidelines and ethics for research with Indigenous populations. This lifelong learning process includes understanding my cultural and spiritual rank, stepping into and living up to cultural roles and responsibilities and drawing upon them during times of need [14].

Lessons learned at my Grandmother’s kitchen table and the kernels of cultural protocol that I now bring with me to my research are all based on one fundamental set of values and principles that has shaped me, and now shapes my research. These values and principles are all underpinned by concepts and components of building relationships. Before ‘stepping out’ into an Aboriginal community as an Aboriginal researcher to interview women and listen to their stories and research their experiences, other cultural protocols need to be attended to. These protocols include establishing support for my research to be undertaken, and also, very importantly, support for me as an individual and for the organisation I work for, to undertake the research.

### Being both insider and outsider

Being Aboriginal in the world of research means many things in many contexts. I am living and working on another Aboriginal people’s country. This requires knowledge of cultural protocols and respect and acknowledgment of those on whose land I have chosen to live, work and now research. As an Aboriginal public health researcher it is important I take consideration of my Aboriginal identity to build trustful and reciprocal relationships in the Aboriginal community in Victoria. In this context I am what some qualitative researchers refer to as both an ‘insider’ and an ‘outsider’ [15]. As an ‘insider’ I have two main roles: Aboriginal community member and Aboriginal researcher in the community. As an ‘outsider’, I am referring to my place of origin. I must acknowledge the country of those on whose land I live, work and research, pay my respects at all times and conduct my research in ways that honour our Aboriginal culture and cultural protocols.

Earning trust, knowing what is happening for the community at different times, and building relationships with Elders, community members, health workers and other stakeholders in my research are essential foundations for going forward with my research. Establishing trustful, respectful and reciprocal relationships with community members, community organisations and other stakeholders is an on-going process that will shape all components of my research from methodology to methods, recruitment to dissemination.

The relationships I have built and will continue to build connect me to community. By being connected to community I am kept informed. By being informed I am held in the same way that I was as a young Aboriginal girl in my Grandmother’s kitchen. I am learning, listening, being told stories and talking with other Aboriginal women. The relationships built throughout this stage of my research connect me to Elders, community members and community organisations, all of which have helped set the pace for my research project. The wisdom of those with whom I have connected in community has continued the learnings in my Grandmother’s kitchen, continuing on our cultural ways of knowing when the time for me is right to begin my research and go forward with cultural and research integrity.

Researching in this way takes time. It is very important that the pace is set by Aboriginal community. This means it is important for researchers to take the time needed to build strong and trustful relationships required for meaningful collaboration in the design, conduct and feeding back of research findings to communities. If protocols are followed ensuring that research is of benefit to community and given back; then researchers can feel confident that there is a place of welcome in Aboriginal community.

## In summary

Research has previously had a “bad name” in Aboriginal communities. Research has too often been viewed as something to gather and take away from Aboriginal people. This paper reflects on the ways that research can be undertaken in respectful, reciprocal ways that involve the Aboriginal community on all levels of the research process beginning with the building of relationships. The call for change to past practices that denied and excluded Aboriginal people from having a voice in the research process has been addressed by the Australian Government in documents such as the NHMRC Guidelines for Ethical Conduct in Aboriginal and Torres Strait Islander Health Research, and Road Map I and II [7,8,12]. These and other resources developed to provide avenues for change, to learn from past mistakes and to map out new approaches are valuable tools for undertaking research with Aboriginal and Torres Strait Islander communities. I look forward with excitement to the next stages of my research using approaches to public health research that include and involve communities. I am living and learning something new every day from this process and being inspired by Elders, community members, my PhD supervision panel and my cultural knowledge. Importantly, I am continuing on the lessons learnt in my Grandmother’s kitchen and finding my ground in public health research.

## Competing interests

The author declares that she has no competing interests.

## Authors' contributions

TK wrote and read the final manuscript.
